# Correlation between Quality and Geographical Origins of *Poria cocos* Revealed by Qualitative Fingerprint Profiling and Quantitative Determination of Triterpenoid Acids

**DOI:** 10.3390/molecules23092200

**Published:** 2018-08-31

**Authors:** Li-Xia Zhu, Jun Xu, Ru-Jing Wang, Hong-Xiang Li, Yu-Zhu Tan, Hu-Biao Chen, Xiao-Ping Dong, Zhong-Zhen Zhao

**Affiliations:** 1 Pharmacy College, Chengdu University of Traditional Chinese Medicine, Chengdu 611137, China; alicesuly@163.com (L.-X.Z.); rujing58@163.com (R.-J.W.); rhinonmr@163.com (H.-X.L.); tanyuzhu@cdutcm.edu.cn (Y.-Z.T.); 2 School of Chinese Medicine, Hong Kong Baptist University, Kowloon, Hong Kong, China; davidxujun@hkbu.edu.hk (J.X.); hbchen@hkbu.edu.hk (H.-B.C.)

**Keywords:** *Poria cocos*, fingerprint, UHPLC-QTOF-MS/MS, UHPLC-QqQ-MS/MS, quantification, multivariate statistical analysis

## Abstract

*Poria cocos* (Schw.) Wolf (PC) is a well-known saprophytic fungus, and its sclerotium without the epidermis (PCS) is widely used in traditional Chinese medicine and as a functional food in many countries. PCS is normally collected from multiple geographical regions, but whether and how the quality of PCS correlates with where it grows have not been determined. This correlation could be significant both for quality control and optimum utilization of PCS as a natural resource. In this study, a qualitative fingerprint profiling method performed by ultra-performance liquid chromatography (UHPLC) with diode array detection (DAD) combining quadrupole time-of-flight-mass spectrometry (QTOF-MS/MS) and a quantitative UHPLC coupled with triple quadrupole mass spectrometry (QqQ-MS/MS) approach were established to investigate whether and how the quality of PCS correlates with its collection location. A standard fingerprint of PCS was generated by median simulation of 25 tested samples collected from four main producing areas of China, and similarity analysis was applied to evaluate the similarities between the fingerprints of samples and the standard fingerprint. Twenty three common peaks occurring in the fingerprint were unequivocally or tentatively identified by UHPLC-QTOF-MS/MS. Meanwhile, principal component analysis (PCA), supervised orthogonal partial least squares-discriminate analysis (OPLS-DA) and hierarchical cluster analysis (HCA) were employed to classify 25 batches of PCS samples into four groups, which were highly consistent with the four geographical regions. Ten compounds were screened out as potential markers to distinguish the quality of PCS. Nine triterpene acids, including five compounds that played important roles in the clusters between different samples collected from the four collection locations, were simultaneously quantified by using the multiple reaction monitoring (MRM) mode of UHPLC-QqQ-MS/MS. The current strategy not only clearly expounded the correlation between quality and geographical origins of PCS, but also provided a fast, accurate and comprehensive qualitative and quantitative method for assessing the quality of PCS.

## 1. Introduction

*Poria cocos* (Schw.) Wolf (PC) is a well-known saprophytic fungus growing around the old and dead roots of several species of pine trees [1]. PC with pine root attached possesses three different medicinal parts, the epidermis, middle part and middle-plus-inner parts, which have long been used for different syndromes or diseases [1]. Of these, the middle part, equivalent to PC sclerotium without the epidermis (PCS), is known as fuling in Chinese. Among the three parts, PCS is the most widely used as both a traditional medicine and a dietary supplement in many countries because of its diuretic, tonic and sedative properties [2]. Nearly 15% of traditional Chinese medicine preparations recorded in the Chinese Pharmacopoeia (2015 edition) comprise PCS. More than 80 triterpenoids have been isolated from PC to date [1,2]. Modern pharmacological research indicates that the triterpenoid acids in PCS, such as pachymic acid (PA), tumulosic acid (TUA), dehydrotumulosic acid (DTUA), poricoic acid A (PAA), poricoic acid B (PAB), polyporenic acid C (PAC), dehydrotrametenolic acid (DTRA), eburicoic acid (EA) and dehydroeburicoic acid (DEA), have a broad range of bioactivities including diuretic [3,4], anti-bacterial [5,6], anti-inflammatory [7,8,9], anti-oxidant [10,11], anti-tumor [12,13,14], anti-hyperglycemic [15], anti-emetic [16] and anti-rejection [17]. These triterpenoid acids are thus commonly regarded as the major bioactive components of PCS and are usually selected as chemical markers for the quality assessment of PCS [1,2].

It is well known that natural medicinal or dietary materials are normally collected from multiple geographical regions. Accumulating studies have demonstrated that place of origin could significantly affect quality of the materials since climate and environment influence biosynthesis and accumulation of secondary metabolites in organism [18]. The origin places of PC are also multiple; in China, PC mainly grows in Yunnan, Hubei, Anhui, Henan and Hunan Provinces [19,20,21]. However, whether and how the quality of PCS differs according to collection location have largely not been determined. Such a correlation, if present, would be significant for both quality control and efficient utilization of PCS; it could also impact artificial cultivation of PCS. Although PCS materials from different locations have been chemically analyzed in some studies [19,20,21], the research aims therein were to test the feasibility of newly-developed analytical assays for PCS, but not to investigate the correlation between collection locations and quality of PCS. Specifically, one study [19] established a qualitative method (HPLC-MS^n^) based on similarity analysis (SA), cluster analysis and principal component analysis (PCA) to determine the quality of different PCS samples, in which collection locations of the PCS samples did not correlate with their quality; moreover, the qualitative fingerprint was developed in a long time (within 80 min). Similarly, another study [21] showed the identification of some triterpenes and the quantification of seven triterpene acids in PC; however, the quantitative analysis was not associated with geographical origins of PC.

Qualitative fingerprint profiling and quantitative determination of bioactive chemicals are promising strategies for quality evaluation of Chinese medicinal materials. However, there is no such qualitative and quantitative method documented in the Chinese Pharmacopoeia (2015 edition) for quality control of PCS. Thus, it is significant to develop a comprehensive, practical and efficient strategy for routine quality assessment of PCS by qualitative and quantitative chemical characterization.

High-performance liquid chromatography (HPLC) coupled with a diode array detection (DAD) detector or an evaporative light scattering detector (ELSD) is the most commonly used to perform qualitative and quantitative characterization [22,23]. However, the conventional HPLC analysis suffers multifaceted disadvantages: long elution time, large solvent consumption, poor resolution and low sensitivity [19,20]. Recently, ultra-high performance liquid chromatography (UHPLC) has been gaining popularity in the fingerprint characterization of traditional Chinese medicine owing to its faster analysis, better separation performance and low solvent consumption [24]. Quadrupole time-of-flight-mass spectrometry (QTOF-MS/MS) is a kind of tandem mass spectrometry providing high mass resolution and accurate mass measurement for structural elucidation of unknown chemicals [25]. In addition, multiple reaction monitoring (MRM) mode operated on triple quadrupole mass spectrometry (QqQ-MS/MS), another kind of tandem mass spectrometry, is a powerful quantitative mode that can achieve high sensitivity based on screening of assigned precursor ion-to-product ion transitions [26,27]. Therefore, UHPLC coupled with QTOF-MS/MS has potential for qualitative fingerprint profiling with higher efficiency and more chemical information, while UHPLC coupled with QqQ-MS/MS is expert in simultaneous quantitative determination of multi-components.

In this study, we investigated whether and how the quality of PCS correlates with its collection location by combining UHPLC-QTOF-MS/MS-based qualitative fingerprint profiling and UHPLC-QqQ-MS/MS-based quantitative determination of triterpenoid acids. First, 25 batches of PCS samples were collected from different regions of China and prepared for analysis. Second, a rapid UHPLC-DAD fingerprint method (within 8 min) was established, and 23 common peaks occurring in the chromatograms were structurally elucidated by QTOF-MS/MS. Third, the data obtained were processed by multivariate statistical analysis, including similarity analysis (SA), principal component analysis (PCA), supervised orthogonal partial least squared discriminant analysis (OPSL-DA) and hierarchical cluster analysis (HCA), to evaluate the differences in quality of these PCS samples. Finally, in order to better understand the differences from quantitative levels, nine major bioactive triterpene acids, including five potential markers that played key roles in differentiating PCS samples from different locations, were simultaneously determined in the PCS samples by using a newly-developed UHPLC-QqQ-MS/MS approach in MRM mode.

## 2. Results and Discussion

### 2.1. Sample Extraction

The extraction procedure was obtained on the basis of our recent research [1]. In the UHPLC fingerprint analysis, the extraction procedure was determined using 10 vols of ethanol (the extraction solvent to sample ratio, *v*/*w*, mL/g) for 60 min at 60 °C in an ultrasonic water bath (300 W). In the quantitative analysis of nine triterpenoid acids by UHPLC-QqQ-MS/MS, the extraction solvent to sample ratio (*v*/*w*, mL/g) was increased to 50:1, and samples were soaked for 30 min at 60 °C before ultra-sonication, so that the nine analytes could be completely extracted.

### 2.2. Establishment of UHPLC Fingerprint

**Figure 1:**
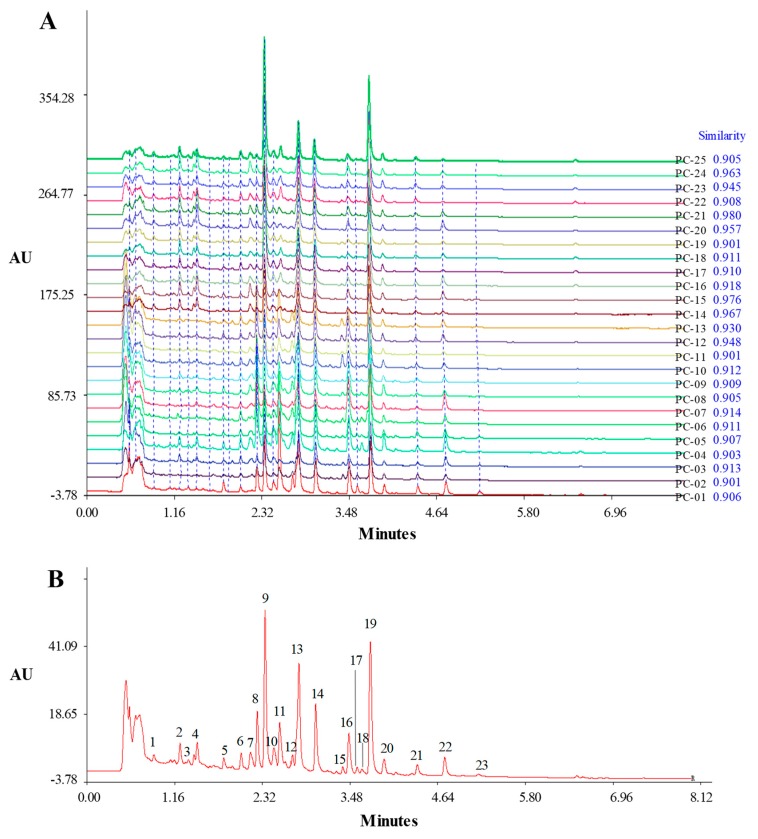
Overlay chromatograms of the 25 *Poria cocos* sclerotium without the epidermis (PCS) samples by SES software and the similarity of each chromatogram to their simulated mean chromatogram in blue (**A**) and the representative standard fingerprint generated by SES software (**B**).

We established a rapid UHPLC-DAD fingerprint rather than a UHPLC-MS fingerprint in order to improve the method universality. The mobile phase system of UHPLC was optimized to develop the fingerprint for PCS. The selection criteria of the procedure included the number of peaks, the resolution between peaks and the separation duration. In order to reduce the ionization and lower the polarity of the triterpenoid acids, a small amount of formic acid was added into the mobile phase. Two organic solvents, acetonitrile and methanol, with different gradient elution conditions were compared. An acetonitrile 0.1% formic acid system with a linear gradient program of 54–82% B in 0–3 min, 82–95% B in 3–6 min, 95–95% B in 6–8 min was chosen for its good resolution and rapid analysis duration (A: 0.1% phosphoric acid aqueous solution, B: acetonitrile). The UV spectrum of each peak in the chromatogram was obtained using the DAD detector. The optimal detection wavelength of 230 nm was selected based on scanning across the whole UV spectrum (200–400 nm), because the largest number of chromatographic peaks with relatively large absorptions appeared in the fingerprint at this wavelength. However, the baseline at the end of the fingerprint was not smooth. Since the non-volatile solvent cannot be used for mass spectrometry, the addition of small amounts of various acids (formic acid, acetic acid and phosphoric acid) was investigated on the UHPLC-DAD instrument. The result demonstrated that using 0.1% phosphoric acid instead of 0.1% formic acid could make the baseline of the chromatographic fingerprint smooth due to its lack of UV absorption at the end, and the chemical profile did not vary. Figure 1A shows the chromatograms of PCS samples under optimized separation conditions. As can be seen, the separation could be completed only within 8 min.

Twenty-five batches of PCS samples collected from four provinces in China were analyzed under the optimized conditions. As shown in Figure 1A, all PCS samples showed similar chromatographic profiles. However, there were obvious differences in peak number and peak absorption intensity between samples. Peaks existing in all samples were designated as “common peaks” for PCS; 23 common peaks were found. The common peaks were further quantitatively expressed in terms of relative retention times (RRT) and relative peak areas (RPA). Peak 20 (pachymic acid) was designated as the reference peak (RRT = retention time of characteristic peak/retention time of reference peak, and RPA = peak area of characteristic peak/peak area of reference peak). The results indicated that the RRT of the 23 common peaks did not vary between samples, which showed that the present UHPLC method is valid for PCS fingerprint analysis and that RRT is a suitable parameter for chemical composition identification. However, the RPA varied dramatically between samples, which demonstrated that the quality of PCS from various locations was different (Supplementary Materials Table S1 and Table S2). It is well known that the standard fingerprint must be representative of the authentic herb. For this purpose, the fingerprints of 25 batches of PCS samples were analyzed by the Similarity Evaluation System for Chromatographic Fingerprint of Traditional Chinese Medicine (SES, Version 2004 A). The software was used to synchronize the fingerprint peaks and to calculate the correlation coefficients between the entire chromatographic profiles and then to generate the representative standard fingerprint by median simulation. Twenty three common peaks appeared in the standard fingerprint (Figure 1B). The similarity between the fingerprint of the tested sample and the standard fingerprint was also analyzed by SES. The similarity of each chromatogram to their simulated mean chromatogram was 0.924 ± 0.026 (mean ± SD, n = 25) (Figure 1A). The similarity demonstrates that the standard fingerprint accurately represents the chemical characteristics of PCS. Therefore, a sample with a similar chromatographic profile and matching RRT values to the standard fingerprint shown in Figure 1B could be authenticated as PCS.

### 2.3. Identification of Common Peaks in the UHPLC Fingerprint

**Figure 2:**
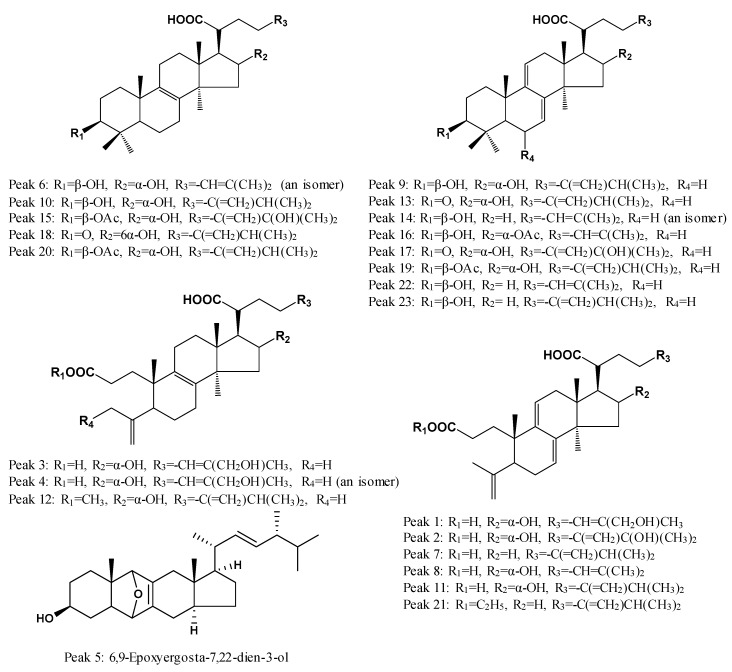
Chemical structures of the compounds identified.

The structural identification of each peak in the UHPLC fingerprint was carried out by the developed UHPLC-QTOF-MS/MS method in both positive and negative ion modes. Twenty three common peaks were characterized by determining accurate molecular mass, generated molecular ions and fragment ions with QTOF-MS/MS and by matching these data with corresponding data of known compounds in our recent study [1], other reported literature and databases. In this way, 22 compounds of these 23 common peaks were identified as triterpenoid acids, and Common Peak 5 was characterized as an ergosterol. Eight of these peaks, namely PAB (Peak 8), DTUA (Peak 9), TUA (Peak 10), PAA (Peak 11), PAC (Peak 13), PA (Peak 20), DTRA (Peak 22) and DEA (Peak 23), were unequivocally identified by comparing their mass data with the mass data of their reference standards. The chemical structures of these compounds are shown in Figure 2. Mass accuracy for the adduct ions was lower than 5 ppm, demonstrating that the empirical molecular formula accurately mapped the putative product ions. The fragmentation pathways of these triterpenoid acids were explained and reported in our recent study [1], so they were not repeated here.

### 2.4. Multivariate Statistical Analysis of QTOF-MS/MS Data

**Figure 3:**
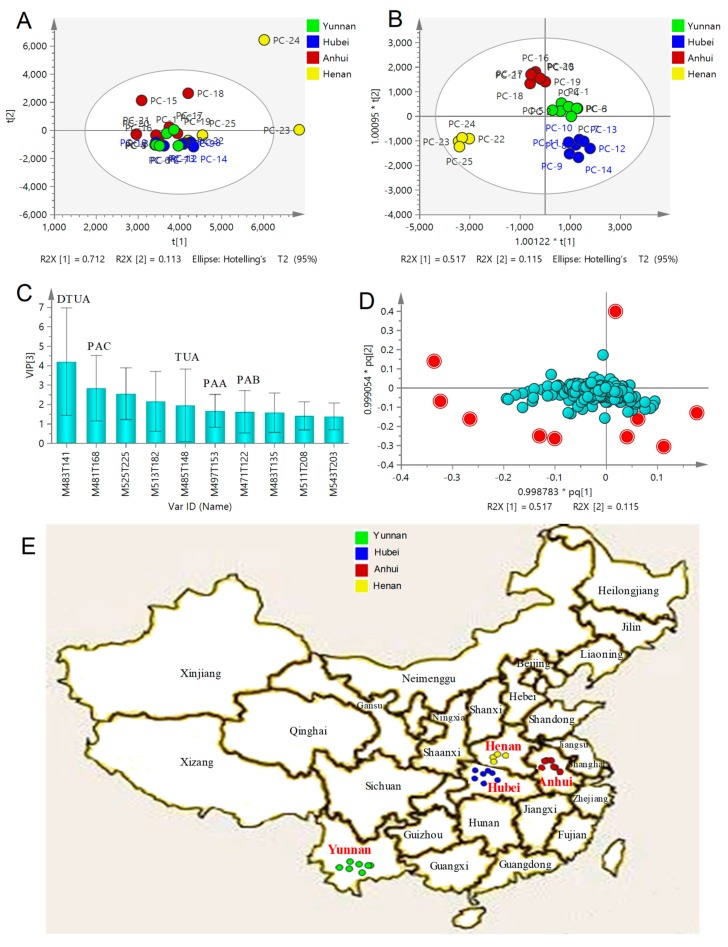
PCA/score plot (**A**), OPLS-DA/score plot (**B**), OPLS-DA/VIP plot (**C**) and OPLS-DA/loading plot (**D**) based on the holistic chemical profiling of 25 PCS samples from different places and the regional distribution of the corresponding 25 PCS samples (**E**). DTUA, dehydrotumulosic acid; PAC, polyporenic acid C; TUA, tumulosic acid; PAA, poricoic acid A; PAB, poricoic acid B. VIP, variable importance for the projection.

In order to explore the dissimilarity among the 25 batches of PCS samples collected from different places, principal component analysis (PCA) and supervised orthogonal partial least squared discriminant analysis (OPSL-DA) were used for processing the chemical profile MS data. PCA, an unbiased multivariate analysis method, was initially applied to investigate whether different sources of PCS samples could be separated according to the differences in chemical compositions. After Pareto scaling, mean-centering and dimensionality reduction, all data were expressed as scores in a coordinate system of principal components. As shown in Figure 3A, the PCA score plot showed that different samples were not completely separated, in which the first principal component accounted for 71.2% of the variance, providing a good PCA model with good reproducibility (R2X = 0.917, close to one) and good predictability (Q2 = 0.741, greater than 0.5).

In order to further characterize the differences in chemical profiles among different PCS samples, OPLS-DA, a supervised latent structures-discriminant analysis technique, which utilizes class information to maximize the separation between classes and minimize the discrimination between intra-groups, was performed to achieve better separation among different samples [28]. The score plot of OPLS-DA indicated that all 25 samples were unambiguously classified into four groups (Figure 3B), thereby demonstrating some chemical dissimilarity in the type and/or content of constituents among different samples. Interestingly, these four groups were highly consistent with the four collection locations of these PCS samples (Figure 3E), which demonstrated that the chemical components of different PCS samples are heavily influenced by growing area. The OPLS-DA model fit parameters were 0.904 of R2X, 0.963 of R2Y and 0.651 of Q2, and all the observations fell within the Hotelling T2 (95%) ellipse, indicating that the developed OPLS-DA model has good fitness and prediction.

In order to reveal which chemical compounds contributed most to the clusters among different places of samples, a variable importance for the projection (VIP) plot and loading plot were constructed in the OPLS-DA model. The VIP plot of OPLS-DA displays the importance of variables both to explain X and to correlate to Y. The OPLS-DA loading plot exhibits the relationship between the X-variables and the Y-variables for the predictive components, and X- and Y-variables with large p or q contribute greatly to the OPLS-DA model. In other words, the further the ion [M − H]^−^ *m*/*z*-RT (s) pair point departs from zero, the greater the ion pair contributes to the clusters among the four groups. Based on the VIP values (i.e., larger than 1.0), 10 triterpenoid acids were observed to play key roles in the clustering (Figure 3C). These 10 compounds, including five chemical standards, corresponded to those ion pair points in red far away from zero in the loading plot of OPLS-DA (Figure 3D).

**Figure 4:**
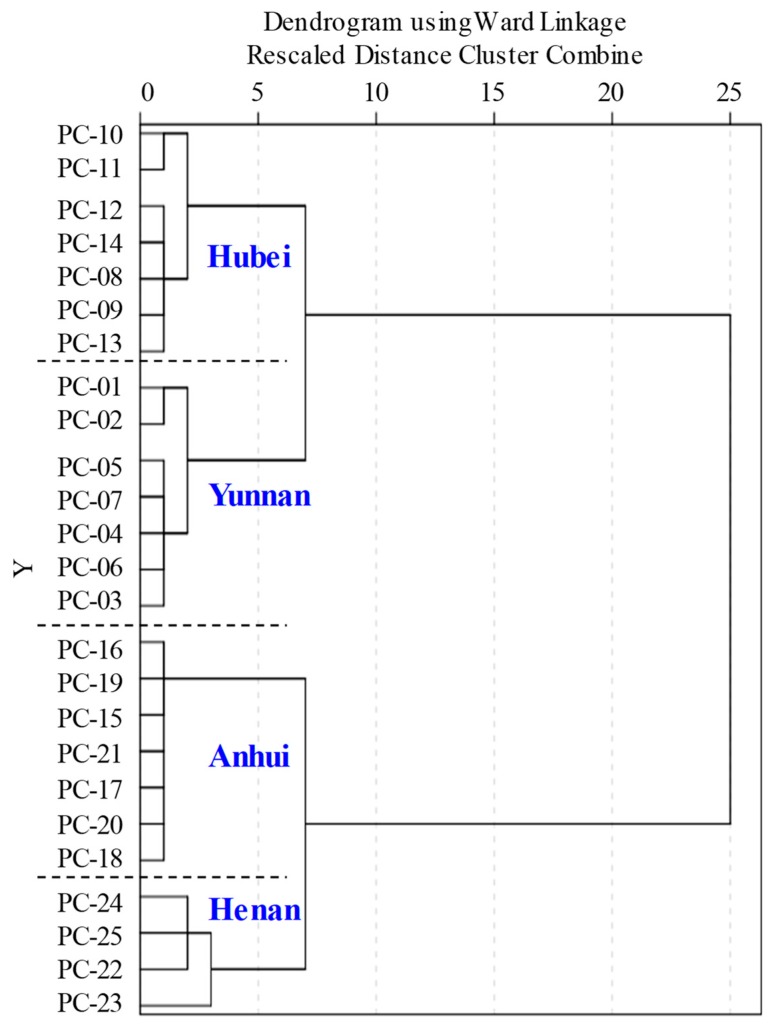
Dendrograms of HCA for 25 PCS samples from different regions.

In addition, HCA was employed to explore the differences in chemical constituents among the four different regions of PCS samples. Ten compounds with variable importance in the VIP plot of OPLS-DA, which served as potential markers, can minimize the noise interference and improve the classification effectiveness, were selected for HCA. The result showed that 25 batches of PCS samples could be mainly divided into four groups consistent with collection locations (Figure 4), further confirming the results of OPLS-DA. Therefore, these 10 compounds could be considered as the most important chemical markers of quality assessment to distinguish between different samples of PCS.

It is worth mentioning that the chemical profile of PCS was found to be different in previous studies [19,20,21]. In addition to analytical conditions, such as extraction conditions, columns and instruments, the place of origin was highly suspected to affect the chemical profile of PCS. In this study, the results of the multivariate statistical analysis did reveal that there was a clear correlation between the quality of PCS and its collection location.

### 2.5. Qualitative and Quantitative Method Validation

A comprehensive validation of the established UHPLC fingerprint method was carried out, including assessments of stability, precision and repeatability, and the results are summarized in Table 1. The intra- and inter-day variations of all 23 common peaks (RSDs) were within 0.02–0.19% and 0.05–0.32% for the RRT, as well as 1.62–4.73% and 1.83–4.87% for the RPA, respectively. In the 48-h stability test, the RSDs of the RRT from all common peaks were lower than 0.28%, and the RSDs of their RPA were less than 4.87%. As for repeatability, the RSDs for the RRT and RPA were not higher than 0.25% and 4.58%, respectively. All these results illustrated that the developed UHPLC fingerprint method was suitable for the qualitative analysis of PCS.

Quantitative method validation for the established UHPLC-QqQ-MS/MS analysis was performed for linearity, intra- and inter-day precision, stability, LODs, lower limits of quantification (LLOQs), repeatability and recovery, as shown in Table 2. All correlation coefficient values (r > 0.995) demonstrated a good linear relationship between the analyte concentrations and their peak areas within the relatively wide test ranges. The intra- and inter-day variations of nine analytes (RSDs) were within 1.01–4.45% and 0.84–6.60%, respectively. The RSDs for stability were not more than 5.97%. The LODs and LLOQs were in the range of 0.37–142 ng/mL and 1.01–458 ng/mL, respectively. As for repeatability, the RSDs were lower than 7.12%. The developed method also had acceptable accuracy with spike recovery of 82.98–108.55% for all analytes. All these results distinctly demonstrated that the proposed quantitative UHPLC-QqQ-MS/MS method was linear, precise, stable, sensitive, repeatable and accurate enough for simultaneous determination of nine triterpenoid acids in PCS samples.

### 2.6. Quantitative Analysis of Triterpenoid Acids

**Figure 5:**
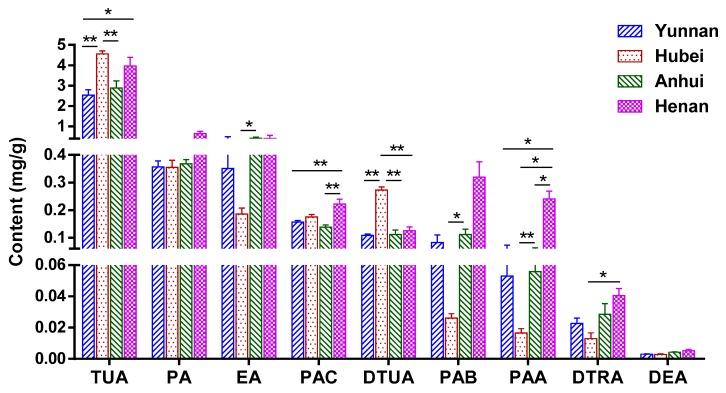
The contents of nine triterpenoid acids in the 25 PCS samples from four regions (* *p* < 0.05 and ** *p* < 0.01).

Nine bioactive triterpenoid acids, namely PAB, DTUA, TUA, PAA, PAC, EA, PA, DTRA and DEA, were selected as the reference standards for quantitation. In our previous study [1], it was found that the triterpenoids detected by UHPLC-QTOF-MS/MS accumulated much less in the middle than in the other two parts, while PCS samples used in the present study are equivalent to the middle part, so UHPLC-QqQ-MS/MS was employed in this study because of its high sensitivity. Twenty five batches of PCS samples from different regions were quantitatively analyzed using the MRM mode of the developed UHPLC-QqQ-MS/MS. As seen from Supplementary Materials Figure S1, MRM chromatograms indicated that the MRM mode provided acceptable chromatographic resolution for these nine analytes. The contents of these analytes in various PCS samples from different places were determined by the above-established UHPLC-QqQ-MS/MS method and then calculated using the constructed calibration curves (Table 2). Only the amounts of PA and DEA had no significant difference among samples from the four collection locations, while the remaining seven analytes displayed obvious dissimilarities. For example, the contents of TUA and DTUA were highest in PCS samples from Hubei province, while lowest in herbs from Yunnan province. The PCS samples from Henan province contained the highest amounts of PAC, PAA and DTRA. Overall, the contents of TUA, being 2.535–4.561 mg/g, were much higher than other analytes in all samples, while DEA was among the lowest. In addition, Graph Pad Prism 6.0 software (GraphPad Software Inc., San Diego, CA, USA) was used for drawing a quantitative diagram. The detailed content trends of nine analytes in the 25 samples from different habitats are exhibited in Figure 5 (bar graph with average content and SEM in the four growing areas) and Supplementary Materials Table S3. These quantitative results of nine bioactive triterpenoid acids provide a valuable reference for differentiating samples collected from different regions and for quality assessment of PCS.

## 3. Materials and Methods

### 3.1. Chemicals and Materials

Twenty-five batches of PCS were collected from four main production regions in China. Of these, seven batches were collected from Yunnan (sample code: PC-01–PC-07), seven from Hubei (sample code: PC-08–PC-14), seven from Anhui (sample code: PC-15–PC-21) and another four from Henan (sample code: PC-22–PC-25). All the herb samples were authenticated with morphological and histological methods by Prof. Zhao Zhongzhen from the School of Chinese Medicine, Hong Kong Baptist University. Voucher specimens were deposited in the Bank of China (Hong Kong) Chinese Medicines Centre of Hong Kong Baptist University.

Pachymic acid (PA), tumulosic acid (TUA), dehydrotumulosic acid (DTUA), poricoic acid A (PAA), poricoic acid B (PAB), polyporenic acid C (PAC), dehydrotrametenolic acid (DTRA, 3β-hydroxylanosta-7,9(11),24-trien-21-oic acid), eburicoic acid (EA) and dehydroeburicoic acid (DEA) were purchased from Chengdu MUST Bio-Technology Co., Ltd. (Chengdu, China), Chengdu PUSH Bio-Technology Co., Ltd. (Chengdu, China) and Shanghai Tauto Biotech Co. Ltd. (Shanghai, China). The purity of each standard compound was above 98%, as determined by high performance liquid chromatography (HPLC) analysis. Acetonitrile (MS grade) was purchased from E. Merck (Darmstadt, Germany). Formic acid was of MS grade from Tedia Company Inc. (Fairfield, OH, USA). Ethanol for extraction was purchased from Merck (Darmstadt, Germany). Deionized water was prepared by a Milli-Q water purification system (Millipore, Bedford, MA, USA).

### 3.2. Samples Preparation

#### 3.2.1. Sample Extraction for Qualitative Analysis

All the herb samples were dried, ground, then sifted through a 24-mesh sieve. Powdered sample (1.0 g), accurately weighed, and 10 mL of ethanol were added into a 50-mL centrifuge tube, weighed together, ultrasonically extracted 60 min in an ultrasonic water bath (300 W) at 60 °C, weighed again and made up to original weight with ethanol, then centrifuged at 1800× *g* for 10 min. The extracted solution was filtered by a 0.22-μm syringe filter for UHPLC fingerprint and identification analysis by UHPLC-PDA-QTOF-MS/MS.

#### 3.2.2. Sample Extraction for Quantitative Analysis

Powdered sample (1.0 g), accurately weighed, and 50 mL of ethanol were added into a 100-mL conical flask, weighed together, soaked for 30 min at 60 °C in an ultrasonic water bath (300 W), ultrasonically extracted 60 min at 60 °C, weighed again and made up to original weight with ethanol, then centrifuged at 1800× *g* for 10 min. The extracted solution was filtered by a 0.22-μm syringe filter for UHPLC-QqQ-MS/MS analysis.

### 3.3. UHPLC Fingerprint Analysis

A rapid UHPLC fingerprint analysis of 25 batches of PCS was achieved by UHPLC-DAD performed on a Waters ACQUITY UPLC system with a diode array detector (Waters Corporation, Milford, MA, USA). The chromatographic separation was performed with a Waters ACQUITY UPLC^®^BEH-C_18_ (2.1 mm × 100 mm, I.D. 1.7 µm) and a VanGuard^TM^ BEH-C_18_ guard column (2.1 mm × 5 mm, I.D. 1.7 µm). The mobile phase consisted of 0.1% phosphoric acid (A) and acetonitrile (B). The linear gradient elution program was optimized as follows: 0–3 min, 54–82% B; 3–6 min, 82–95% B; 6–9 min, 95–95% B. The flow rate was set at 0.40 mL/min. The column was maintained at 40 °C. The injection volume was 2 μL. The optimal detection wavelength was determined by scanning across the whole UV spectrum (200–400 nm).

### 3.4. UHPLC-DAD-QTOF-MS/MS Analysis

Common peaks in the UHPLC fingerprint were characterized by UHPLC-QTOF-MS/MS. UHPLC was performed on an Agilent 1290 Infinity system (Agilent Technologies Inc., Palo Alto, CA, USA) equipped with an auto-sampler and binary solvent delivery system. The same UPLC C_18_ analytical column was used and maintained at the same temperature of 40 °C as in UHPLC fingerprint analysis. The mobile phase consisted of 0.1% formic acid (A) and acetonitrile (B). The linear gradient elution condition, injection volume and flow rate were the same as in UHPLC fingerprint analysis.

Mass spectrometry was performed on an Agilent 6540 ultra-high definition accurate mass quadrupole time-of-flight spectrometer (Agilent Technologies Inc., Wilmington, DE, USA). The mass spectra were acquired both in the positive and negative mode by scanning from 100–1700 in a mass-to-charge ratio (*m*/*z*). The mass spectrometry analysis was performed under the following optimized conditions: dry gas temperature 300 °C, dry nitrogen gas flow rate 8 L/min, nebulizer pressure 40 psi, Vcap 4500, nozzle voltage 500 V and fragmentor voltage 150 V. The collision energies were set at 15 and 35 eV. ESI-Low concentration tuning mix (Agilent Technologies, Part No. G1969-85000) was used for mass calibration. This tune mix covered the mass ranges from 118–1521 at *m*/*z* (positive mode) and 112–1634 at *m*/*z* (negative mode). Calibration was performed every day to ensure mass accuracy.

The data processing software Agilent MassHunter Workstation and Q-TOF Qualitative Analysis (Version B.06.00, Agilent Technologies Inc., 2012) were used to identify chromatographic peaks.

### 3.5. UHPLC-QqQ-MS/MS Analysis

Quantitative analysis of nine triterpenoid acids was achieved by UHPLC-QqQ-MS/MS. The UHPLC instrument was the same as in the above UHPLC-QTOF-MS/MS analysis. The chromatographic separation was performed with the same UPLC C_18_ analytical column as in UHPLC fingerprint analysis. The mobile phase consisted of water (A) and acetonitrile (B), both of which contained 0.1% formic acid. The linear gradient elution condition was optimized as follows: 0–7.5 min, 5–80% B; 7.5–8.5 min, 80–100% B. The flow rate was set at 0.40 mL/min. The column was maintained at 20 °C. The injection volume was 3 μL.

Mass spectrometry was performed on an Agilent 6460 QqQ/MS system equipped with an electrospray ionization (ESI) source. The conditions of the ESI source were as follows: drying gas (N2) flow rate, 8.0 L/min; drying gas temperature, 350 °C; nebulizer pressure, 45 psi; capillary voltage, 3500 V (+) and 4000 V (−); nozzle voltage, 500 V; dwell time, 20 min. The quantitative analysis was performed using MRM mode, and the detailed MRM conditions for each analyte are given in Supplementary Materials Table S4. The cycle time for all MRM transitions is 503 ms/cycle, and the cycle time already includes the pause time between MRM transitions and the switching time between positive and negative mode. The acquisition rate for this method is thus 1.99 cycle/s (around 2 data points per second). The peak widths of nine analytes are around 9–12 s. Around 18–24 data points can be acquired across a peak, which is good enough for quantitation. Agilent MassHunter Quantitative Analysis Software B.04.00 (Agilent Technologies Inc., Santa Clara, CA, USA) was used to collect and process mass data.

### 3.6. Qualitative and Quantitative Method Validation

The developed UHPLC-QqQ-MS/MS method for quantitative analysis of nine major triterpenoid acids was validated under the above-described optimized conditions in terms of linearity, precision, stability, repeatability, accuracy and sensitivity.

A series of standard solutions for constructing working standard curves was prepared by diluting the mixed stock standard solution with ethanol, and a calibration curve was established by plotting peak areas (y axis) versus concentrations (x axis). The levels of concentration in the linearity study were: 0.5, 2, 10, 20, 40, 60 and 100 µg/mL for TUA and EA; 0.25, 1, 5, 10, 20, 30 and 50 µg/mL for PA; 0.125, 0.5, 2.5, 5, 10, 15 and 25 µg/mL for DTUA and PAC; 0.1, 0.4, 2, 4, 8, 12 and 20 µg/mL for PAB; 0.05, 0.2, 1, 2, 4, 6 and 10 µg/mL for PAA, DEA and DTRA.

A mixed standard solution was analyzed for six replicates within the same day and additionally on three consecutive days for evaluating intra- and inter-day precision, respectively. For the stability assessment, the extract of a sample (PC-02) was analyzed at 0, 2, 4, 8, 12, 24 and 48 h at room temperature. Repeatability was evaluated by analyzing six sample solutions prepared individually from the same batch of samples (PC-02). The recovery test was conducted to assess the accuracy of the method by spiking the sample (PC-02) with different levels (low, middle and high) of known contents of reference compounds in the sample, with nine independent spiked analyses in totality. The limits of detection (LODs) and lower limits of quantification (LLOQs) were determined by continuously diluting the standard solution until the S/N (signal to noise) ratios reached around 3 and 10, respectively.

The established UHPLC fingerprint method was validated in terms of intra- and inter-day precision, stability and repeatability using a similar above-described method, but the precision was determined by injecting the same sample solution (PC-02). Common peaks in the PCS fingerprint were chosen to calculate their RRT and RPA.

### 3.7. Statistical Analysis

PCA and OPSL-DA were applied to characterize the dissimilarities in chemical components among different samples with regard to collection locations. The raw QTOF-MS/MS data of 25 samples from four main geographical regions in negative ion mode were processed by first using DA Reprocessor (Version B.06.00, Agilent Technologies Inc., 2012) with the following settings: height filtering with minimum absolute height, 3000 counts; peak spacing tolerance with a maximum of 0.0025 *m*/*z* plus 7.0 ppm; limit assigned charge states to a maximum of 2. Then, relative quantification and comparison of small molecule compounds’ profiles after the above-processed UHPLC-QTOF-MS/MS data were performed with XCMS data analysis software [28]. PCA and OPSL-DA were performed using the software SIMCA-P Version 13.0 (Umetrics, Umeå, Sweden). From the variable importance for the projection (VIP) plot and loading plot of OPLS-DA, various chemical components could be extracted as being dominantly responsible for discriminating between different regions of PCS samples. HCA, one-way ANOVA and the Welch test were conducted by IBM SPSS Statistics 21.0 software (IBM, Armonk, NY, USA). Statistical differences among contents of 9 triterpenoid acids in PCS samples from different collection locations were assessed by one-way ANOVA followed by the Scheffe test for multiple comparisons if the variance was homogeneous, while the Welch test followed by Dunnett’s T3 test for multiple comparisons was adopted if the variance was not homogeneous. 

## 4. Conclusions

In this study, a method combining UHPLC-QTOF-MS/MS-based qualitative fingerprint profiling and UHPLC-QqQ-MS/MS-based quantitative determination of nine bioactive triterpene acids was developed and validated for exploring the correlation between collection locations and quality of PCS. Twenty three peaks found in all 25 samples from different habitats were regarded as common peaks, and all were characterized by UHPLC-QTOF-MS/MS. A standard fingerprint for PCS was proposed based on median simulation of all tested samples and analysis of the similarities between the fingerprints of different samples and the proposed standard fingerprint. Then, PCA, OPLS-DA and HCA were applied to investigate the dissimilarities among the chemical components in PCS samples collected from different places. Twenty five batches of PCS samples were clearly classified into four groups, corresponding to the four collection locations. Furthermore, 10 triterpene acids were found to be chemical markers that could be used to discriminate the internal quality of PCS. Nine bioactive triterpenoid acids, including five compounds that contributed significantly to the clusters of samples from different regions, were simultaneously quantified by using the MRM mode of UHPLC-QqQ-MS/MS. The current study revealed a clear correlation between quality and geographical region of origin. According to the correlation, the raw materials of PCS from different regions can be formulated in a certain proportion and used for its traditional Chinese medicine preparations, thereby ensuring the safety, stability and effectiveness of clinical application; however, extensive pharmacological research is still required to address this issue. In a word, the proposed fingerprint will be important for authentication; the developed quantitative method will be useful for quality control of PCS and its preparations; the revealed correlation could be significant for optimum utilization of PCS as a natural resource.

## Figures and Tables

**Table 1:** The proposed UHPLC-DAD method validation of common peaks in the PCS fingerprint. RRT, relative retention times; RPA, relative peak areas.

Peak No.	Precision (RSD, %)				Stability (RSD, %)		Repeatability (RSD, %)	
	Intra-Day		Inter-Day					
	RRT	RPA	RRT	RPA	RRT	RPA	RRT	RPA
1	0.02	3.26	0.08	4.58	0.06	4.87	0.03	3.31
2	0.04	4.73	0.18	3.92	0.21	4.09	0.09	4.19
3	0.09	2.69	0.15	4.03	0.14	3.84	0.06	2.80
4	0.15	4.28	0.24	3.85	0.17	4.72	0.13	2.94
5	0.05	1.97	0.07	3.14	0.09	2.51	0.03	1.79
6	0.02	2.88	0.10	2.36	0.08	3.11	0.06	3.53
7	0.18	3.95	0.26	4.87	0.20	4.39	0.09	3.65
8	0.03	3.42	0.14	1.83	0.06	0.96	0.04	2.48
9	0.07	2.16	0.05	3.11	0.12	1.57	0.08	1.85
10	0.10	4.71	0.16	2.94	0.12	4.63	0.13	3.64
11	0.19	3.02	0.32	4.12	0.28	2.85	0.25	2.39
12	0.06	4.13	0.20	3.47	0.15	3.68	0.19	2.54
13	0.05	2.40	0.08	3.78	0.17	1.25	0.10	3.08
14	0.03	1.62	0.07	3.15	0.09	2.94	0.07	2.81
15	0.07	3.89	0.10	2.99	0.05	4.12	0.08	1.42
16	0.04	2.35	0.12	3.44	0.06	1.67	0.03	0.86
17	0.08	1.91	0.05	3.80	0.03	3.34	0.11	4.13
18	0.02	2.96	0.08	2.69	0.03	4.58	0.07	4.58
19	0.03	3.45	0.05	1.92	0.02	2.53	0.07	0.62
20	0.00	0.00	0.00	0.00	0.00	0.00	0.00	0.00
21	0.04	2.67	0.10	3.15	0.02	1.80	0.06	1.56
22	0.06	3.46	0.17	2.74	0.03	2.36	0.12	2.24
23	0.11	3.82	0.16	4.07	0.07	4.74	0.06	3.50

**Table 2:** The established UHPLC-QqQ-MS/MS method validation for quantitative determination of nine triterpenoid acids. PA, pachymic acid; DTRA, dehydrotrametenolic acid; DEA, dehydroeburicoic acid; EA, eburicoic acid; LLOQs, lower limits of quantification.

Reference Standards	Working Standard Curve			Precision (RSD, %, *n* = 6)		Stability (48 h, RSD, %)	Sensitivity (ng/mL)		Repeatability (RSD, %, *n* = 6)	Spike Recovery (Mean (RSD), %, *n* = 3)		
	Equation	r	Linear Range (µg/mL)	Intra-Day	Inter-Day		LODs	LLOQs		Low	Middle	High
PAB	y = 139,887x + 104,903	0.9968	0.1–20	1.84	3.48	2.88	0.45	1.18	2.27	94.72 (7.38)	101.65 (5.63)	96.88 (6.24)
DTUA	y = 32,635x + 170,109	0.9967	0.125–25	3.25	5.49	3.35	0.74	2.23	5.35	93.59 (8.26)	98.32 (4.08)	105.16 (7.50)
TUA	y = 784x + 75,827	0.9959	0.5–100	3.62	3.71	2.92	1.83	4.86	3.91	104.35 (2.78)	99.49 (1.82)	96.95 (6.63)
PAA	y = 129,115x − 11,973	0.9946	0.05–10	4.45	4.13	4.47	0.37	1.01	4.18	97.86 (3.43)	104.91 (4.85)	92.64 (5.74)
PAC	y = 11,879x + 44,483	0.9957	0.125–25	2.14	4.77	3.75	0.49	1.54	3.64	102.48 (6.89)	98.76 (3.91)	107.19 (8.03)
PA	y = 63,136x + 576,258	0.9998	0.25–50	1.01	4.71	1.81	0.58	1.82	1.70	96.42 (4.07)	100.54 (2.58)	103.42 (3.98)
DTRA	y = 23,506x + 25,923	0.9956	0.05–10	2.67	0.84	3.36	3.04	8.79	6.39	86.75 (9.11)	93.63 (6.87)	105.38 (8.56)
DEA	y = 10,217x + 125	0.9996	0.05–10	3.29	6.60	4.85	0.97	2.65	7.12	82.98 (10.85)	106.26 (8.36)	108.55 (7.25)
EA	y = 283x − 4	0.9989	0.5–100	4.25	4.38	5.97	142	458	5.69	92.14 (6.75)	96.93 (7.84)	106.63 (5.27)

## Supplementary Materials

The following are available online: Figure S1: Typical MRM chromatograms by UHPLC-QqQ-MS/MS of reference standards (A) and a representative sample (PC-02, B) for quantitation; Table S1: RRT of common peaks in 25 batches of PCS samples; Table S2: RPA of common peaks in 25 batches of PCS samples; Table S3: Contents of nine triterpenoid acids in 25 samples from four main geographical regions of PCS; Table S4: Optimized MRM conditions for nine triterpenoid acids.
