# Supplementary material for: Correlation between Quality and Geographical Origins of Poria cocos Revealed by Qualitative Fingerprint Profiling and Quantitative Determination of Triterpenoid Acids
Source: Molecules. 2018 Aug 31;23(9):2200. doi: 10.3390/molecules23092200 (PMC6225149; doi:10.3390/molecules23092200)
Supplement: Supplementary file 1 [file molecules-23-02200-s001.zip › Supplementary files/Supplementary Materials Tables.docx]

**Legends of Supplementary Tables**

**Table S1 RRT of common peaks in 25 batches of PCS samples.**

**Table S2 RPA of common peaks in 25 batches of PCS samples.**

**Table S3 Contents of nine triterpenoid acids in 25 samples from four main geographical regions of PCS.**

**Table S4 Optimized MRM conditions for nine triterpenoid acids.**

**Table S1 RRT of common peaks in 25 batches of PCS samples.**

| Peak no. | Sample code | | | | | | | | | | | | | | | | | | | | | | | | | Mean | RSD (%) |
| --- | --- | --- | --- | --- | --- | --- | --- | --- | --- | --- | --- | --- | --- | --- | --- | --- | --- | --- | --- | --- | --- | --- | --- | --- | --- | --- | --- |
|  | PC-01 | PC-02 | PC-03 | PC-04 | PC-05 | PC-06 | PC-07 | PC-08 | PC-09 | PC-10 | PC-11 | PC-12 | PC-13 | PC-14 | PC-15 | PC-16 | PC-17 | PC-18 | PC-19 | PC-20 | PC-21 | PC-22 | PC-23 | PC-24 | PC-25 |  |  |
| 1 | 0.22 | 0.23 | 0.23 | 0.23 | 0.23 | 0.23 | 0.23 | 0.23 | 0.23 | 0.23 | 0.23 | 0.23 | 0.23 | 0.23 | 0.22 | 0.23 | 0.22 | 0.22 | 0.23 | 0.22 | 0.23 | 0.23 | 0.23 | 0.22 | 0.23 | 0.23 | 0.27 |
| 2 | 0.31 | 0.31 | 0.31 | 0.31 | 0.31 | 0.31 | 0.31 | 0.31 | 0.31 | 0.31 | 0.31 | 0.31 | 0.31 | 0.31 | 0.32 | 0.31 | 0.31 | 0.32 | 0.31 | 0.31 | 0.31 | 0.31 | 0.31 | 0.32 | 0.31 | 0.31 | 0.52 |
| 3 | 0.34 | 0.34 | 0.34 | 0.34 | 0.34 | 0.34 | 0.34 | 0.34 | 0.34 | 0.34 | 0.34 | 0.34 | 0.34 | 0.34 | 0.34 | 0.34 | 0.34 | 0.34 | 0.34 | 0.34 | 0.34 | 0.34 | 0.34 | 0.34 | 0.34 | 0.34 | 0.39 |
| 4 | 0.37 | 0.37 | 0.37 | 0.37 | 0.37 | 0.37 | 0.37 | 0.37 | 0.37 | 0.37 | 0.37 | 0.37 | 0.37 | 0.37 | 0.37 | 0.37 | 0.37 | 0.37 | 0.37 | 0.37 | 0.37 | 0.37 | 0.37 | 0.37 | 0.37 | 0.37 | 0.33 |
| 5 | 0.46 | 0.46 | 0.46 | 0.46 | 0.46 | 0.46 | 0.46 | 0.46 | 0.46 | 0.46 | 0.46 | 0.46 | 0.46 | 0.46 | 0.46 | 0.46 | 0.46 | 0.46 | 0.46 | 0.46 | 0.46 | 0.46 | 0.46 | 0.46 | 0.46 | 0.46 | 0.23 |
| 6 | 0.52 | 0.52 | 0.52 | 0.52 | 0.52 | 0.52 | 0.52 | 0.52 | 0.52 | 0.52 | 0.52 | 0.52 | 0.52 | 0.52 | 0.52 | 0.52 | 0.52 | 0.52 | 0.52 | 0.52 | 0.52 | 0.52 | 0.52 | 0.52 | 0.52 | 0.52 | 0.23 |
| 7 | 0.55 | 0.55 | 0.55 | 0.55 | 0.55 | 0.55 | 0.55 | 0.55 | 0.55 | 0.55 | 0.55 | 0.55 | 0.55 | 0.55 | 0.56 | 0.55 | 0.55 | 0.56 | 0.56 | 0.55 | 0.55 | 0.54 | 0.55 | 0.54 | 0.55 | 0.55 | 0.49 |
| 8 | 0.57 | 0.57 | 0.57 | 0.57 | 0.57 | 0.57 | 0.57 | 0.57 | 0.57 | 0.57 | 0.57 | 0.57 | 0.57 | 0.57 | 0.57 | 0.57 | 0.57 | 0.57 | 0.57 | 0.57 | 0.57 | 0.57 | 0.57 | 0.57 | 0.57 | 0.57 | 0.16 |
| 9 | 0.60 | 0.60 | 0.60 | 0.60 | 0.60 | 0.60 | 0.60 | 0.60 | 0.60 | 0.60 | 0.60 | 0.60 | 0.60 | 0.60 | 0.60 | 0.60 | 0.60 | 0.60 | 0.60 | 0.60 | 0.60 | 0.60 | 0.60 | 0.60 | 0.60 | 0.60 | 0.22 |
| 10 | 0.63 | 0.63 | 0.63 | 0.63 | 0.63 | 0.63 | 0.63 | 0.63 | 0.63 | 0.63 | 0.63 | 0.63 | 0.63 | 0.63 | 0.63 | 0.63 | 0.63 | 0.63 | 0.63 | 0.63 | 0.63 | 0.63 | 0.63 | 0.63 | 0.63 | 0.63 | 0.25 |
| 11 | 0.65 | 0.65 | 0.65 | 0.65 | 0.65 | 0.65 | 0.65 | 0.66 | 0.66 | 0.66 | 0.66 | 0.66 | 0.66 | 0.66 | 0.65 | 0.65 | 0.65 | 0.65 | 0.65 | 0.65 | 0.65 | 0.65 | 0.65 | 0.65 | 0.65 | 0.65 | 0.54 |
| 12 | 0.69 | 0.69 | 0.69 | 0.69 | 0.69 | 0.69 | 0.69 | 0.69 | 0.69 | 0.69 | 0.69 | 0.69 | 0.69 | 0.69 | 0.69 | 0.69 | 0.69 | 0.69 | 0.69 | 0.69 | 0.69 | 0.69 | 0.69 | 0.69 | 0.69 | 0.69 | 0.25 |
| 13 | 0.71 | 0.71 | 0.71 | 0.71 | 0.72 | 0.72 | 0.72 | 0.71 | 0.71 | 0.71 | 0.72 | 0.72 | 0.72 | 0.72 | 0.71 | 0.71 | 0.71 | 0.71 | 0.71 | 0.71 | 0.71 | 0.71 | 0.71 | 0.71 | 0.71 | 0.71 | 0.22 |
| 14 | 0.77 | 0.77 | 0.77 | 0.77 | 0.77 | 0.77 | 0.77 | 0.77 | 0.77 | 0.77 | 0.77 | 0.77 | 0.77 | 0.77 | 0.77 | 0.77 | 0.77 | 0.77 | 0.77 | 0.77 | 0.77 | 0.77 | 0.77 | 0.77 | 0.77 | 0.77 | 0.09 |
| 15 | 0.86 | 0.86 | 0.86 | 0.86 | 0.86 | 0.86 | 0.86 | 0.86 | 0.86 | 0.86 | 0.86 | 0.86 | 0.86 | 0.86 | 0.86 | 0.86 | 0.86 | 0.86 | 0.86 | 0.86 | 0.86 | 0.86 | 0.86 | 0.86 | 0.86 | 0.86 | 0.05 |
| 16 | 0.88 | 0.88 | 0.88 | 0.88 | 0.88 | 0.88 | 0.88 | 0.88 | 0.88 | 0.88 | 0.88 | 0.88 | 0.88 | 0.88 | 0.88 | 0.88 | 0.88 | 0.88 | 0.88 | 0.88 | 0.88 | 0.88 | 0.88 | 0.88 | 0.88 | 0.88 | 0.07 |
| 17 | 0.91 | 0.91 | 0.91 | 0.91 | 0.91 | 0.91 | 0.91 | 0.91 | 0.91 | 0.91 | 0.91 | 0.91 | 0.91 | 0.91 | 0.91 | 0.91 | 0.91 | 0.91 | 0.91 | 0.91 | 0.91 | 0.91 | 0.91 | 0.91 | 0.91 | 0.91 | 0.03 |
| 18 | 0.93 | 0.93 | 0.93 | 0.93 | 0.93 | 0.93 | 0.93 | 0.93 | 0.93 | 0.92 | 0.93 | 0.93 | 0.93 | 0.93 | 0.93 | 0.92 | 0.93 | 0.93 | 0.93 | 0.93 | 0.93 | 0.92 | 0.93 | 0.93 | 0.93 | 0.93 | 0.03 |
| 19 | 0.95 | 0.95 | 0.95 | 0.95 | 0.95 | 0.95 | 0.95 | 0.95 | 0.95 | 0.95 | 0.95 | 0.95 | 0.95 | 0.95 | 0.95 | 0.95 | 0.95 | 0.95 | 0.95 | 0.95 | 0.95 | 0.95 | 0.95 | 0.95 | 0.95 | 0.95 | 0.03 |
| 20 | 1.00 | 1.00 | 1.00 | 1.00 | 1.00 | 1.00 | 1.00 | 1.00 | 1.00 | 1.00 | 1.00 | 1.00 | 1.00 | 1.00 | 1.00 | 1.00 | 1.00 | 1.00 | 1.00 | 1.00 | 1.00 | 1.00 | 1.00 | 1.00 | 1.00 | 1.00 | 0.00 |
| 21 | 1.11 | 1.11 | 1.11 | 1.11 | 1.11 | 1.11 | 1.11 | 1.11 | 1.11 | 1.11 | 1.11 | 1.11 | 1.11 | 1.11 | 1.11 | 1.11 | 1.11 | 1.11 | 1.11 | 1.11 | 1.11 | 1.11 | 1.11 | 1.11 | 1.11 | 1.11 | 0.04 |
| 22 | 1.20 | 1.20 | 1.20 | 1.20 | 1.20 | 1.20 | 1.20 | 1.20 | 1.20 | 1.20 | 1.20 | 1.20 | 1.20 | 1.20 | 1.21 | 1.21 | 1.21 | 1.21 | 1.21 | 1.20 | 1.20 | 1.21 | 1.21 | 1.21 | 1.21 | 1.20 | 0.13 |
| 23 | 1.32 | 1.32 | 1.32 | 1.32 | 1.31 | 1.32 | 1.32 | 1.32 | 1.32 | 1.32 | 1.32 | 1.32 | 1.32 | 1.32 | 1.32 | 1.32 | 1.32 | 1.32 | 1.32 | 1.32 | 1.32 | 1.32 | 1.32 | 1.32 | 1.32 | 1.32 | 0.17 |

**Table S2 RPA of common peaks in 25 batches of PCS samples.**

| Peak no. | Sample code | | | | | | | | | | | | | | | | | | | | | | | | | Mean | RSD (%) |
| --- | --- | --- | --- | --- | --- | --- | --- | --- | --- | --- | --- | --- | --- | --- | --- | --- | --- | --- | --- | --- | --- | --- | --- | --- | --- | --- | --- |
|  | PC-01 | PC-02 | PC-03 | PC-04 | PC-05 | PC-06 | PC-07 | PC-08 | PC-09 | PC-10 | PC-11 | PC-12 | PC-13 | PC-14 | PC-15 | PC-16 | PC-17 | PC-18 | PC-19 | PC-20 | PC-21 | PC-22 | PC-23 | PC-24 | PC-25 |  |  |
| 1 | 1.45 | 1.79 | 2.71 | 1.61 | 3.33 | 3.17 | 2.08 | 1.33 | 1.83 | 3.18 | 2.53 | 2.00 | 1.57 | 1.53 | 2.46 | 2.42 | 1.55 | 1.27 | 1.28 | 2.20 | 1.29 | 0.64 | 0.47 | 1.44 | 0.70 | 1.83 | 42.22 |
| 2 | 1.33 | 2.04 | 3.03 | 2.67 | 4.47 | 4.19 | 3.25 | 1.94 | 2.29 | 4.56 | 3.07 | 2.56 | 1.99 | 2.03 | 0.56 | 2.88 | 0.92 | 0.50 | 1.77 | 1.58 | 1.64 | 0.46 | 0.96 | 0.64 | 0.89 | 2.09 | 58.20 |
| 3 | 0.80 | 1.06 | 1.42 | 0.89 | 1.51 | 1.49 | 1.09 | 1.13 | 1.04 | 1.49 | 1.10 | 0.96 | 0.92 | 1.06 | 0.81 | 1.21 | 0.47 | 0.71 | 0.95 | 0.64 | 1.99 | 0.64 | 0.41 | 1.04 | 0.65 | 1.02 | 35.73 |
| 4 | 0.92 | 1.07 | 3.54 | 4.06 | 5.81 | 5.34 | 5.13 | 1.85 | 2.31 | 3.52 | 2.52 | 2.39 | 2.07 | 1.74 | 0.75 | 1.84 | 0.54 | 1.22 | 1.14 | 0.99 | 0.96 | 0.76 | 0.86 | 1.04 | 0.94 | 2.13 | 73.32 |
| 5 | 1.03 | 1.48 | 0.80 | 0.54 | 1.04 | 1.19 | 0.88 | 0.72 | 0.91 | 1.29 | 0.68 | 0.89 | 0.94 | 0.55 | 0.96 | 1.35 | 0.58 | 0.77 | 0.82 | 0.74 | 1.25 | 1.08 | 0.41 | 1.15 | 0.86 | 0.92 | 29.55 |
| 6 | 1.18 | 2.02 | 0.74 | 0.48 | 1.02 | 1.24 | 0.76 | 1.56 | 1.36 | 1.59 | 1.20 | 1.32 | 1.05 | 1.48 | 1.02 | 1.85 | 0.85 | 1.23 | 1.54 | 0.83 | 0.97 | 0.71 | 0.72 | 1.79 | 0.55 | 1.16 | 35.69 |
| 7 | 1.28 | 1.76 | 2.25 | 2.08 | 2.84 | 2.70 | 2.63 | 1.59 | 1.70 | 2.60 | 1.74 | 1.84 | 1.78 | 1.34 | 1.21 | 2.43 | 0.59 | 1.54 | 2.02 | 1.05 | 1.66 | 0.24 | 1.13 | 0.71 | 1.05 | 1.67 | 40.81 |
| 8 | 4.11 | 5.48 | 1.19 | 1.68 | 1.91 | 2.34 | 1.71 | 1.22 | 1.19 | 1.38 | 0.92 | 1.16 | 1.29 | 0.86 | 2.03 | 2.05 | 3.07 | 4.78 | 4.11 | 3.15 | 3.66 | 2.58 | 5.34 | 10.59 | 3.15 | 2.84 | 75.19 |
| 9 | 4.36 | 6.37 | 9.54 | 5.88 | 7.84 | 9.44 | 6.70 | 14.40 | 17.47 | 21.35 | 18.70 | 17.28 | 12.42 | 15.33 | 5.19 | 13.64 | 5.49 | 3.84 | 10.69 | 5.11 | 5.01 | 5.93 | 3.10 | 5.08 | 2.82 | 9.32 | 58.66 |
| 10 | 1.59 | 1.87 | 2.54 | 1.48 | 2.33 | 2.40 | 1.82 | 1.88 | 2.39 | 2.70 | 2.51 | 2.41 | 1.86 | 1.84 | 1.45 | 2.59 | 1.49 | 1.67 | 1.97 | 1.15 | 2.46 | 1.55 | 1.94 | 2.46 | 1.92 | 2.01 | 21.62 |
| 11 | 3.03 | 5.42 | 1.84 | 1.80 | 0.67 | 2.27 | 0.64 | 2.14 | 2.42 | 3.01 | 2.07 | 2.63 | 2.33 | 2.15 | 2.38 | 1.73 | 2.19 | 2.80 | 3.45 | 2.22 | 3.19 | 8.81 | 3.91 | 6.65 | 3.01 | 2.91 | 60.61 |
| 12 | 1.36 | 2.54 | 0.59 | 0.52 | 1.00 | 1.26 | 0.62 | 0.60 | 0.67 | 0.78 | 0.54 | 0.65 | 0.68 | 0.49 | 1.45 | 0.68 | 0.60 | 1.42 | 1.42 | 1.48 | 2.42 | 1.79 | 1.01 | 2.29 | 1.39 | 1.13 | 54.74 |
| 13 | 6.88 | 8.13 | 7.30 | 7.34 | 8.40 | 9.54 | 7.54 | 6.36 | 7.35 | 8.67 | 7.45 | 7.18 | 6.73 | 5.40 | 4.49 | 8.90 | 6.53 | 6.94 | 7.55 | 5.98 | 6.69 | 8.53 | 5.53 | 8.78 | 6.30 | 7.22 | 16.71 |
| 14 | 4.05 | 4.66 | 4.82 | 3.97 | 5.88 | 5.74 | 4.81 | 3.43 | 4.17 | 5.09 | 4.21 | 3.76 | 4.22 | 2.94 | 3.53 | 5.59 | 3.95 | 3.45 | 5.17 | 4.28 | 3.66 | 4.07 | 2.63 | 3.85 | 2.90 | 4.19 | 20.62 |
| 15 | 0.74 | 1.09 | 0.02 | 0.01 | 0.10 | 0.37 | 0.01 | 0.17 | 0.37 | 0.30 | 0.36 | 0.28 | 0.45 | 0.04 | 0.62 | 0.57 | 0.17 | 0.28 | 0.28 | 1.55 | 0.44 | 0.02 | 0.01 | 0.56 | 0.28 | 0.36 | 99.78 |
| 16 | 3.57 | 2.78 | 1.40 | 1.68 | 2.02 | 2.26 | 1.85 | 1.28 | 1.10 | 1.45 | 1.27 | 1.09 | 1.14 | 1.04 | 2.49 | 2.37 | 2.92 | 4.93 | 2.86 | 2.61 | 2.89 | 3.30 | 2.29 | 5.79 | 3.12 | 2.38 | 49.85 |
| 17 | 0.39 | 0.37 | 0.36 | 0.21 | 0.29 | 0.41 | 0.34 | 0.31 | 0.28 | 0.38 | 0.26 | 0.29 | 0.33 | 0.23 | 0.70 | 0.36 | 0.55 | 0.36 | 0.46 | 0.37 | 0.11 | 0.80 | 0.27 | 0.54 | 0.18 | 0.37 | 41.72 |
| 18 | 0.48 | 0.26 | 0.14 | 0.20 | 0.21 | 0.24 | 0.20 | 0.24 | 0.16 | 0.16 | 0.15 | 0.22 | 0.15 | 0.18 | 0.20 | 0.19 | 0.40 | 0.57 | 0.37 | 0.20 | 0.30 | 0.24 | 0.48 | 0.87 | 0.33 | 0.29 | 58.87 |
| 19 | 7.35 | 7.04 | 9.53 | 7.94 | 8.39 | 8.67 | 8.47 | 9.75 | 11.14 | 13.08 | 10.34 | 12.23 | 10.15 | 10.76 | 7.23 | 9.17 | 9.02 | 6.75 | 8.51 | 6.91 | 7.91 | 5.39 | 5.90 | 6.46 | 6.30 | 8.58 | 22.96 |
| 20 | 1.00 | 1.00 | 1.00 | 1.00 | 1.00 | 1.00 | 1.00 | 1.00 | 1.00 | 1.00 | 1.00 | 1.00 | 1.00 | 1.00 | 1.00 | 1.00 | 1.00 | 1.00 | 1.00 | 1.00 | 1.00 | 1.00 | 1.00 | 1.00 | 1.00 | 1.00 | 0.00 |
| 21 | 0.64 | 0.63 | 0.72 | 0.45 | 0.62 | 0.86 | 0.68 | 0.63 | 0.73 | 0.96 | 0.71 | 0.72 | 0.79 | 0.55 | 1.16 | 0.85 | 1.05 | 0.64 | 0.86 | 0.71 | 0.30 | 0.99 | 0.51 | 0.51 | 0.40 | 0.71 | 28.85 |
| 22 | 1.20 | 1.29 | 0.65 | 1.46 | 2.24 | 1.18 | 0.84 | 1.49 | 0.38 | 1.40 | 0.79 | 0.24 | 0.56 | 0.25 | 1.12 | 2.45 | 1.45 | 3.05 | 1.04 | 0.90 | 0.61 | 1.59 | 1.28 | 1.20 | 0.64 | 1.17 | 57.20 |
| 23 | 0.24 | 0.25 | 0.06 | 0.09 | 0.13 | 0.08 | 0.05 | 0.13 | 0.07 | 0.13 | 0.09 | 0.09 | 0.09 | 0.06 | 0.27 | 0.27 | 0.34 | 0.15 | 0.27 | 0.21 | 0.17 | 0.47 | 0.09 | 0.14 | 0.13 | 0.16 | 64.30 |

**Table S3 Contents of nine triterpenoid acids in 25 samples from four main geographical regions of PCS.**

| **Sample code** | | **PAB** | **DTUA** | **TUA** | **PAA** | **PAC** | **EA** | **PA** | **DTRA** | **DEA** |
| --- | --- | --- | --- | --- | --- | --- | --- | --- | --- | --- |
|  | PC-01 | 0.176^a)^ (1.26)^b)^ | 0.0863 (4.23) | 2.06 (3.44) | 0.0960 (2.77) | 0.169 (0.72) | 0.750 (1.44) | 0.436 (1.26) | 0.0305 (2.64) | 0.00442 (5.01) |
|  | PC-02 | 0.196 (3.02) | 0.113 (3.46) | 3.61 (3.67) | 0.153 (4.06) | 0.151 (2.99) | 1.00 (2.08) | 0.402 (2.66) | 0.0256 (5.09) | 0.00465 (5.56) |
|  | PC-03 | 0.0243 (4.33) | 0.118 (1.23) | 2.62 (5.66) | 0.0170 (6.63) | 0.135 (5.15) | 0.101 (3.65) | 0.289 (2.09) | 0.00899 (7.54) | 0.00214 (0.82) |
| **Yunnan** | PC-04 | 0.0605 (1.32) | 0.115 (3.07) | 1.94 (2.91) | 0.0380 (2.55) | 0.167 (4.56) | 0.120 (3.93) | 0.405 (1.03) | 0.0298 (0.65) | 0.00251 (3.39) |
|  | PC-05 | 0.0345 (3.15) | 0.103 (0.67) | 2.01 (3.96) | 0.0165 (2.67) | 0.150 (2.63) | 0.149 (4.81) | 0.297 (3.31) | 0.0320 (1.35) | 0.00263 (4.61) |
|  | PC-06 | 0.0497 (0.47) | 0.122 (2.20) | 3.45 (5.17) | 0.0313 (5.06) | 0.169 (1.49) | 0.246 (4.15) | 0.328 (1.61) | 0.0172 (5.05) | 0.00245 (3.68) |
|  | PC-07 | 0.0405 (0.34) | 0.0998 (1.20) | 2.05 (3.81) | 0.0188 (6.63) | 0.161 (3.46) | 0.0891 (4.54) | 0.342 (2.75) | 0.0150 (5.23) | 0.00172 (5.84) |
| Mean ± SEM | | 0.0830 ± 0.0270 | 0.108 ± 0.00469 | 2.54 ± 0.271 | 0.0529 ± 0.0197 | 0.157 ± 0.00486 | 0.351 ± 0.140 | 0.357 ± 0.0217 | 0.0227 ± 0.00339 | 0.00293 ±0.000430 |
|  | PC-08 | 0.0357 (2.86) | 0.306 (4.79) | 4.99 (3.59) | 0.0197 (4.14) | 0.195 (5.46) | 0.208 (4.82) | 0.391 (0.34) | 0.0336 (4.09) | 0.00487 (2.88) |
|  | PC-09 | 0.0251 (2.95) | 0.293 (3.09) | 4.95 (4.53) | 0.0149 (5.69) | 0.184 (5.34) | 0.187 (4.36) | 0.378 (1.64) | 0.00705 (5.55) | 0.00224 (3.78) |
|  | PC-10 | 0.0161 (5.98) | 0.222 (4.30) | 4.05 (2.39) | 0.00969 (5.14) | 0.132 (5.66) | 0.197 (4.22) | 0.251 (1.66) | 0.0164 (1.59) | 0.00212 (4.09) |
| **Hubei** | PC-11 | 0.0198 (4.16) | 0.249 (3.77) | 4.53 (3.11) | 0.0133 (3.88) | 0.148 (2.30) | 0.245 (3.92) | 0.269 (2.42) | 0.0113 (3.56) | 0.00224 (3.12) |
|  | PC-12 | 0.0262 (1.87) | 0.290 (1.48) | 4.43 (4.98) | 0.0145 (4.48) | 0.191 (0.53) | 0.158 (5.35) | 0.380 (1.70) | 0.00507 (4.55) | 0.00216 (5.25) |
|  | PC-13 | 0.0362 (1.87) | 0.254 (5.89) | 4.80 (3.65) | 0.0317 (2.39) | 0.201 (1.86) | 0.233 (4.32) | 0.432 (2.57) | 0.0111 (5.14) | 0.00315 (3.05) |
|  | PC-14 | 0.0233 (2.88) | 0.293 (3.72) | 4.19 (3.20) | 0.0120 (3.24) | 0.173 (1.52) | 0.076 (5.23) | 0.385 (3.21) | 0.0064 (4.46) | 0.00280 (2.92) |
| Mean ± SEM | | 0.0261 ± 0.00286 | 0.272 ± 0.01165 | 4.56 ± 0.138 | 0.0165 ± 0.0028 | 0.175 ± 0.00972 | 0.186 ± 0.021 | 0.355 ± 0.0256 | 0.0130 ± 0.00372 | 0.00280 ± 0.000375 |
|  | PC-15 | 0.0620 (2.89) | 0.0802 (4.72) | 2.39 (4.89) | 0.0538 (0.78) | 0.0944 (5.39) | 0.345 (3.15) | 0.357 (1.65) | 0.0197 (1.99) | 0.00482 (4.49) |
|  | PC-16 | 0.0424 (3.11) | 0.170 (3.56) | 4.23 (4.58) | 0.0190 (2.38) | 0.146 (1.28) | 0.320 (9.36) | 0.302 (1.08) | 0.0329 (3.13) | 0.00412 (2.15) |
|  | PC-17 | 0.131 (3.07) | 0.107 (2.86) | 2.21 (2.57) | 0.0529 (0.87) | 0.143 (2.08) | 0.309 (4.09) | 0.407 (4.72) | 0.0307 (4.69) | 0.00507 (3.08) |
| **Anhui** | PC-18 | 0.197 (5.27) | 0.0708 (4.04) | 1.96 (3.21) | 0.0780 (3.77) | 0.130 (2.65) | 0.410 (4.98) | 0.403 (0.95) | 0.0655 (2.45) | 0.00498 (1.08) |
|  | PC-19 | 0.126 (6.17) | 0.173 (5.31) | 4.14 (4.36) | 0.0579 (5.92) | 0.150 (5.39) | 0.421 (2.92) | 0.347 (2.79) | 0.0174 (2.49) | 0.00470 (3.76) |
|  | PC-20 | 0.111 (3.19) | 0.0984 (2.78) | 2.64 (3.76) | 0.0591 (3.13) | 0.159 (2.54) | 0.723 (4.82) | 0.407 (0.99) | 0.0199 (4.76) | 0.00377 (1.88) |
|  | PC-21 | 0.114 (1.35) | 0.0815 (1.13) | 2.61 (4.27) | 0.0703 (1.70) | 0.144 (1.79) | 0.400 (1.13) | 0.352 (0.17) | 0.0141 (3.22) | 0.00245 (5.89) |
| Mean ± SEM | | 0.112 ± 0.0190 | 0.112 ± 0.0162 | 2.88 ± 0.348 | 0.0559 ± 0.00705 | 0.138 ± 0.00799 | 0.419 ± 0.0535 | 0.368 ± 0.0150 | 0.0286 ± 0.00667 | 0.00427 ± 0.000353 |
| **Henan** | PC-22 | 0.162 (1.16) | 0.161 (4.91) | 5.14 (4.18) | 0.327 (0.98) | 0.193 (2.29) | 1.01 (3.64) | 0.497 (3.45) | 0.0458 (4.23) | 0.00890 (4.07) |
|  | PC-23 | 0.464 (2.50) | 0.153 (3.85) | 4.48 (2.43) | 0.267 (1.82) | 0.287 (2.39) | 0.056 (5.99) | 1.02 (1.46) | 0.0550 (2.35) | 0.00455 (3.28) |
|  | PC-24 | 0.423 (2.88) | 0.0991 (3.41) | 3.69 (1.29) | 0.212 (2.68) | 0.180 (4.04) | 0.402 (1.32) | 0.411 (3.11) | 0.0276 (4.36) | 0.00340 (3.24) |
|  | PC-25 | 0.231 (2.37) | 0.0905 (3.35) | 2.52 (5.33) | 0.157 (0.73) | 0.229 (3.97) | 0.143 (5.24) | 0.690 (2.52) | 0.0333 (4.55) | 0.00387 (2.87) |
| Mean ± SEM | | 0.320 ± 0.0553 | 0.126 ± 0.0137 | 3.96 ± 0.426 | 0.241 ± 0.0277 | 0.222 ± 0.0182 | 0.402 ± 0.162 | 0.656 ± 0.103 | 0.0404 ± 0.00467 | 0.00518 ± 0.000954 |

1. The data was present as average of triplicate determination (mg/g); ^b)^ The RSD value of triplicate quantitative result (%).

**Table S4 Optimized MRM conditions for nine triterpenoid acids.**

| **Markers** | **RT (min)** | **Precursor Ion** | **Product Ion Ⅰ** | | **Product Ion Ⅱ** | | **Fragmentor Voltage (V)** |
| --- | --- | --- | --- | --- | --- | --- | --- |
|  |  | *m/z* | *m/z* | Collision Energy (V) | *m/z* | Collision Energy (V) |  |
| PAB | 3.05 | 483.3 [M-H]^-^ | 411.3 [M-H-C_3_H_4_O_2_]^-^ | 17 | 409.3 [M-H-CH_3_CH_2_COOH]^-^ | 25 | 212 |
| DTUA | 3.18 | 485.4 [M+H]^+^ | 467.3 [M+H-H_2_O]^+^ | 9 | 311.3 [M+H-H_2_O-C_9_H_16_O_2_]^+^ | 17 | 98 |
| TUA | 3.36 | 485.4 [M-H]^-^ | 485.4 [M-H]^-^ | 5 | 423.2 [M-H-H_2_O-CO_2_]^-^ | 37 | 250 |
| PAA | 3.58 | 497.3 [M-H]^-^ | 425.0 [M-H-C_3_H_4_O_2_]^-^ | 17 | 423.3 [M-H-CH_3_CH_2_COOH]^-^ | 25 | 250 |
| PAC | 3.95 | 481.3 [M-H]^-^ | 421.3 [M-H-CH_3_COOH]^-^ | 29 | 97.1 [M-H-C_25_H_36_O_3_]^-^ | 41 | 250 |
| EA | 4.21 | 469.4 [M-H]^-^ | 371.0 [M-H-C_7_H_14_]^-^ | 45 | 85.2 [M-H-C_25_H_36_O_3_]^-^ | 21 | 250 |
| PA | 6.01 | 529.4 [M+H]^+^ | 511.4 [M+H-H_2_O]^+^ | 9 | 451.4 [M+H-H_2_O-CH_3_COOH]^+^ | 13 | 98 |
| DTRA | 6.99 | 455.4 [M+H]^+^ | 437.4 [M+H-H_2_O]^+^ | 9 | 119.1 [M+H-C_21_H_36_O_3_]^+^ | 49 | 136 |
| DEA | 7.65 | 467.3 [M-H]^-^ | 339.2 [M-H-C_8_H_16_O]^-^ | 25 | 337.3 [M-H-C_7_H_14_O_2_]^-^ | 29 | 250 |
