# Supplementary figures and images for: Correlation between Quality and Geographical Origins of Poria cocos Revealed by Qualitative Fingerprint Profiling and Quantitative Determination of Triterpenoid Acids
Source: Molecules. 2018 Aug 31;23(9):2200. doi: 10.3390/molecules23092200 (PMC6225149; doi:10.3390/molecules23092200)

**A**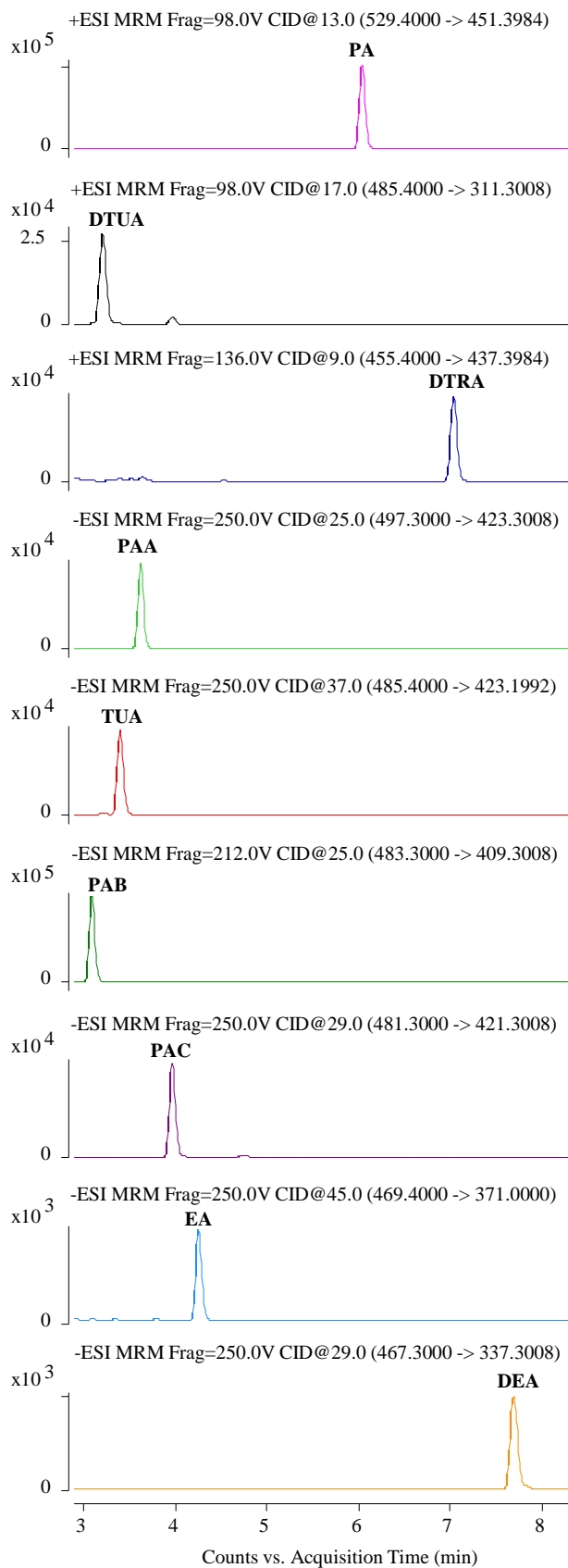**B**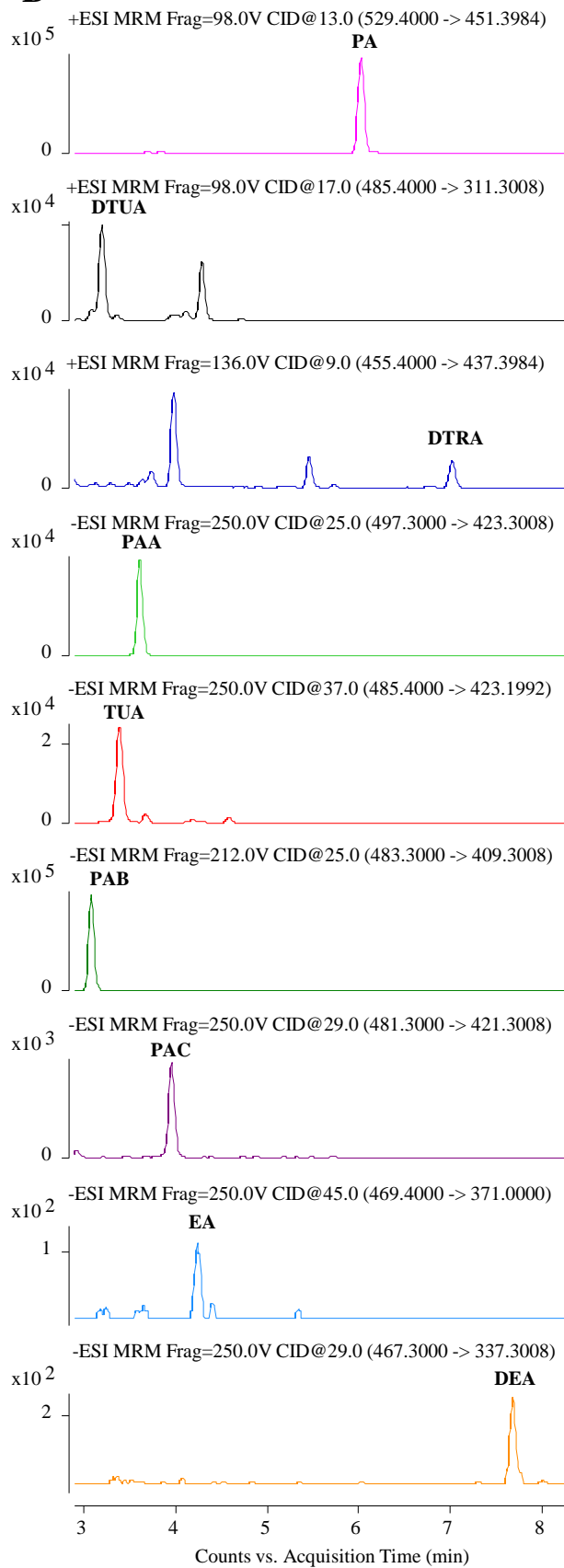

Supplement: Supplementary file 1 [file molecules-23-02200-s001.zip › Supplementary files/Figures S1.pdf]
